# Low-Rank Based Image Analyses for Pathological MR Image Segmentation and Recovery

**DOI:** 10.3389/fnins.2019.00333

**Published:** 2019-04-09

**Authors:** Chuanlu Lin, Yi Wang, Tianfu Wang, Dong Ni

**Affiliations:** National-Regional Key Technology Engineering Laboratory for Medical Ultrasound, Guangdong Provincial Key Laboratory of Biomedical Measurements and Ultrasound Imaging, Health Science Center, School of Biomedical Engineering, Shenzhen University, Shenzhen, China

**Keywords:** MR brain images, image recovery, tumor segmentation, structured sparsity, low-rank, matrix decomposition

## Abstract

The presence of pathologies in magnetic resonance (MR) brain images causes challenges in various image analysis areas, such as registration, atlas construction and atlas-based segmentation. We propose a novel method for the simultaneous recovery and segmentation of pathological MR brain images. Low-rank and sparse decomposition (LSD) approaches have been widely used in this field, decomposing pathological images into (1) low-rank components as recovered images, and (2) sparse components as pathological segmentation. However, conventional LSD approaches often fail to produce recovered images reliably, due to the lack of constraint between low-rank and sparse components. To tackle this problem, we propose a transformed low-rank and structured sparse decomposition (TLS^2^D) method. The proposed TLS^2^D integrates the structured sparse constraint, LSD and image alignment into a unified scheme, which is robust for distinguishing pathological regions. Furthermore, the well recovered images can be obtained using TLS^2^D with the combined structured sparse and computed image saliency as the adaptive sparsity constraint. The efficacy of the proposed method is verified on synthetic and real MR brain tumor images. Experimental results demonstrate that our method can effectively provide satisfactory image recovery and tumor segmentation.

## 1. Introduction

Automated image computing routines (e.g., segmentation, registration, atlas construction) that can analyze the magnetic resonance (MR) brain tumor scans are of essential importance for improved disease diagnosis, treatment planning and follow-up of individual patients (Iglesias and Sabuncu, 2015; Mai et al., 2015; Menze et al., 2015; Chen et al., 2018). Lately, a wave of deep learning is taking over traditional computer aided diagnosis techniques, by learning abundant multi-level features from large amount of training repository for image representation and analyzing (Litjens et al., 2017; Shen et al., 2017). Various architectures of deep convolutional neural networks have been developed and employed for brain tumor segmentation (Pereira et al., 2016; Havaei et al., 2017; Kamnitsas et al., 2017; Zhao et al., 2018). Despite achieving satisfactory performance, deep learning based approaches require enormous amount of labeled images to train a segmentation model. Collecting and labeling useful training samples may last a lengthy duration thus sometimes is clinically impractical. In addition, the presence of pathologies in MR brain images causes difficulties in most of other image analyses, such as image registration, atlas construction and atlas-based anatomical segmentation. The recovery of pathological regions with normal brain appearances can facilitate subsequent image computing procedures. For example, the recovered images could further be used for atlas construction and specific patient's follow-up (Joshi et al., 2004; Liu et al., 2014; Zheng et al., 2017; Han et al., 2018). However, there is lack of deep learning based methods developed for pathological medical image recovery. In contrast, the low-rank and sparse decomposition (LSD) (Wright et al., 2009; Candès et al., 2011) scheme, learning normal image appearance from unlabeled population data, has been widely employed to decompose pathological MR brain images into recovered normal brain appearances and pathological regions (Liu et al., 2015; Tang et al., 2018).

Although the low-rank and sparse analyses of computational brain tumor segmentation has attracted considerable attention during last decade, it remains several challenges. First, conventional LSD methods have to be computed on a series of aligned images (Otazo et al., 2015; Tang et al., 2018), because the image misalignment causes undesired structure differences that would interfere the representation of sparse component. Thus, the image alignment should be conducted before/during the LSD computation; however, the image alignment itself is a challenging task. Second, specific spatial constraint should be imposed on sparse component to restrict the structured sparsity of the tumor region in the whole image. Third, LSD methods often produce recovered images (i.e., low-rank component) with distorted pathological regions (Liu et al., 2015), due to the lack of effective constraint between low-rank and sparse components. Thus it is essential to adaptively balance the low-rank and sparse components to reliably recover tumor regions meanwhile retaining normal brain regions.

**Figure 1:**
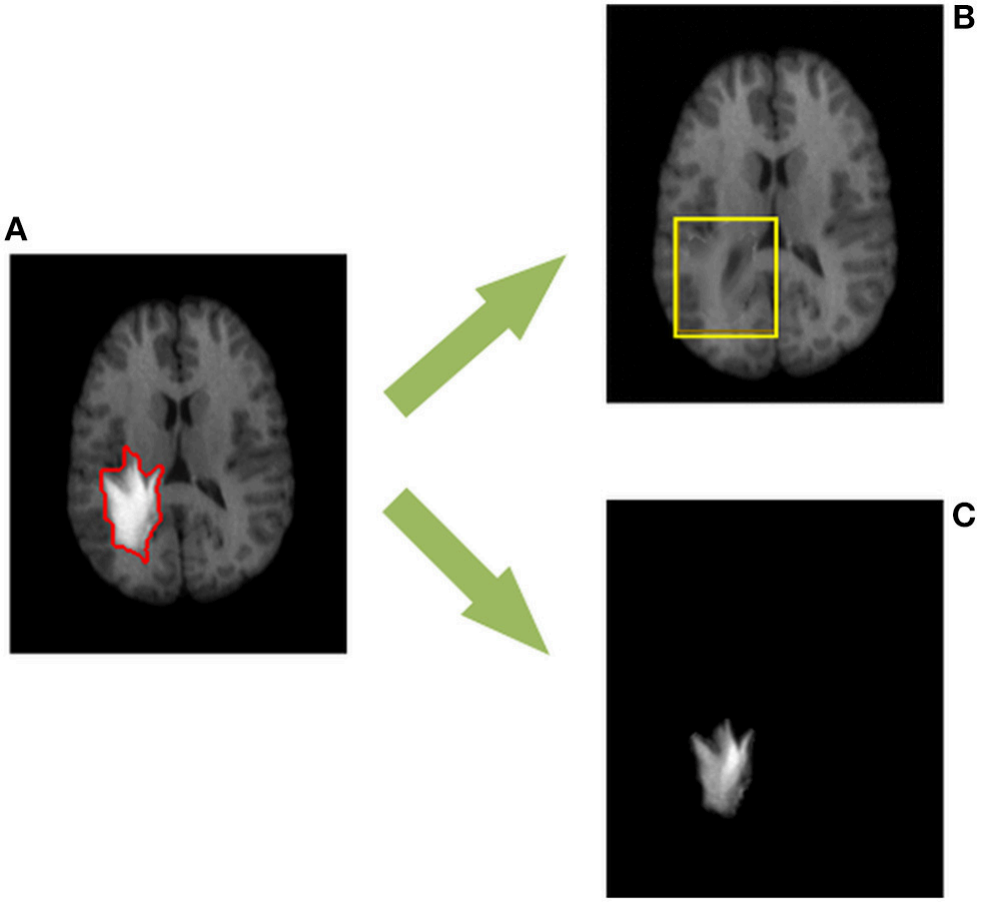
The proposed TLS^2^D method can decompose **(A)** the MR brain tumor image into **(B)** the recovered MR image with quasi-normal brain appearances, and **(C)** the extracted tumor region. The red contour in **(A)** indicates the manually delineated tumor boundary. The yellow box in **(B)** indicates the reliably recovered region.

To address aforementioned issues, this paper presents a novel method for the simultaneous recovery and segmentation of pathological MR brain images (see Figure 1). Specifically, we propose a transformed low-rank and structured sparse decomposition (TLS^2^D) method. The proposed TLS^2^D integrates the structured sparsity constraint, LSD and image alignment into a unified framework, which is robust for extracting pathological regions. Furthermore, the well recovered images can be obtained using TLS^2^D with the combined structured sparse and computed image saliency as the adaptive sparsity constraint. Experimental results on synthetic and real MR brain tumor images demonstrate that the proposed TLS^2^D can effectively extract and recover tumor regions.

## 2. Methods

**Figure 2:**
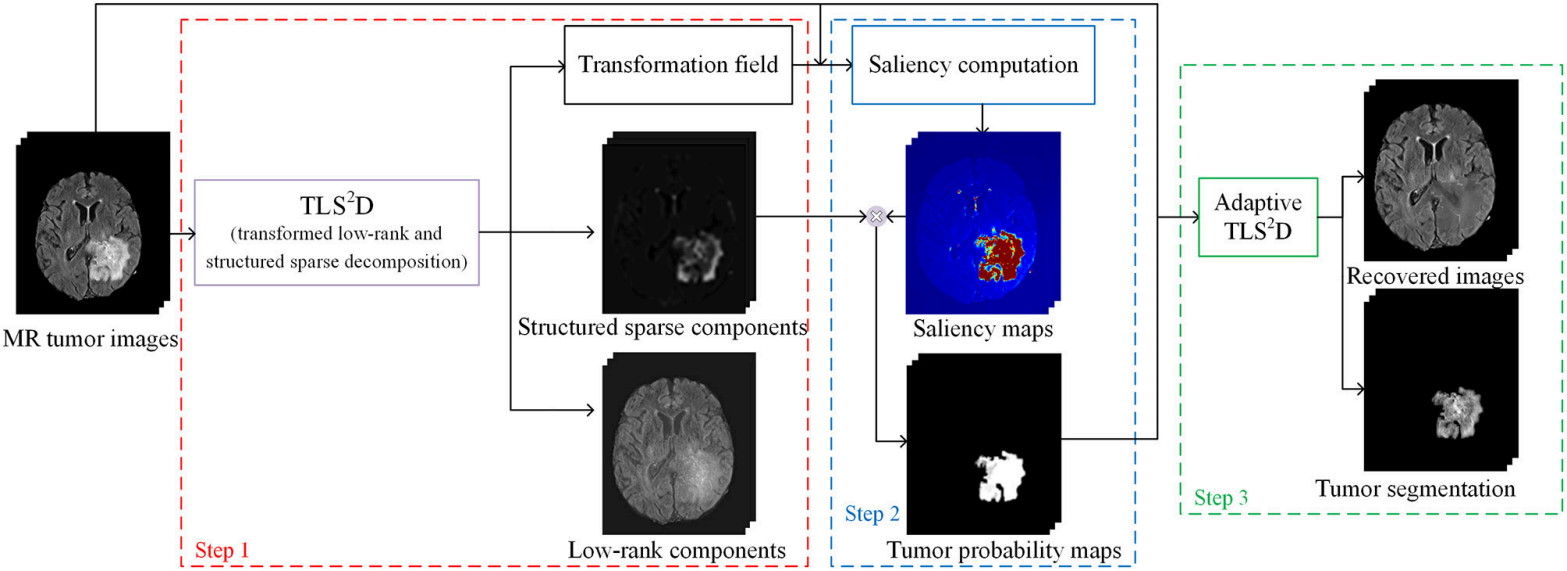
The illustration of the whole recovery and segmentation framework using the proposed transformed low-rank and structured sparse decomposition (TLS^2^D) method.

The proposed recovery and segmentation framework is shown in Figure 2. Our TLS^2^D first iteratively aligns all images and decomposes aligned images into low-rank and structured sparse components. Then the structured sparse components are combined with the computed saliency maps to generate tumor probability maps as the adaptive sparsity constraint. The final recovery and segmentation is obtained by imposing the adaptive sparsity constraint on the TLS^2^D.

The following subsections present a brief review of classical LSD, the details of our method and elaborate the novel TLS^2^D.

### 2.1. Review of Low-Rank and Sparse Decomposition (LSD)

Suppose we are given *n* previously aligned MR brain images $A_{1},A_{2},\ldots ,A_{n}\in {\mathbb{R}}^{w\times h}$, where *w* and *h* denotes width and height of the image, respectively. We can vectorize each image matrix *A*_*n*_ to form the column of $A=\left[vec\left(A_{1}\right),vec\left(A_{2}\right),\ldots ,vec\left(A_{n}\right)\right]\in {\mathbb{R}}^{m\times n}$, where *m* = *w* × *h*.

The conventional LSD method decomposes *A* into a low-rank matrix *L* and a sparse matrix *S*, where *L* indicates the linearly correlated normal images, and *S* represents sparse tumor regions. The decomposition can be solved by the following convex optimization:

(1) $$\begin{array}{l} \underset{L,S}{\min}\;||L|{|}_{*}+\lambda ||S|{|}_{1}\;s.t.\;A=L+S, \end{array}$$

where ||*L*||_*_ is the nuclear norm of *L* (i.e., the sum of its singular values), ||*S*||_1_ is the ℓ_1_ norm of *S*, and regularizing parameter λ weights the relationship between low-rank and sparse components. The optimization in Equation (1) can be solved by augmented Lagrangian multiplier (ALM) method (Lin et al., 2010).

**Figure 3:**
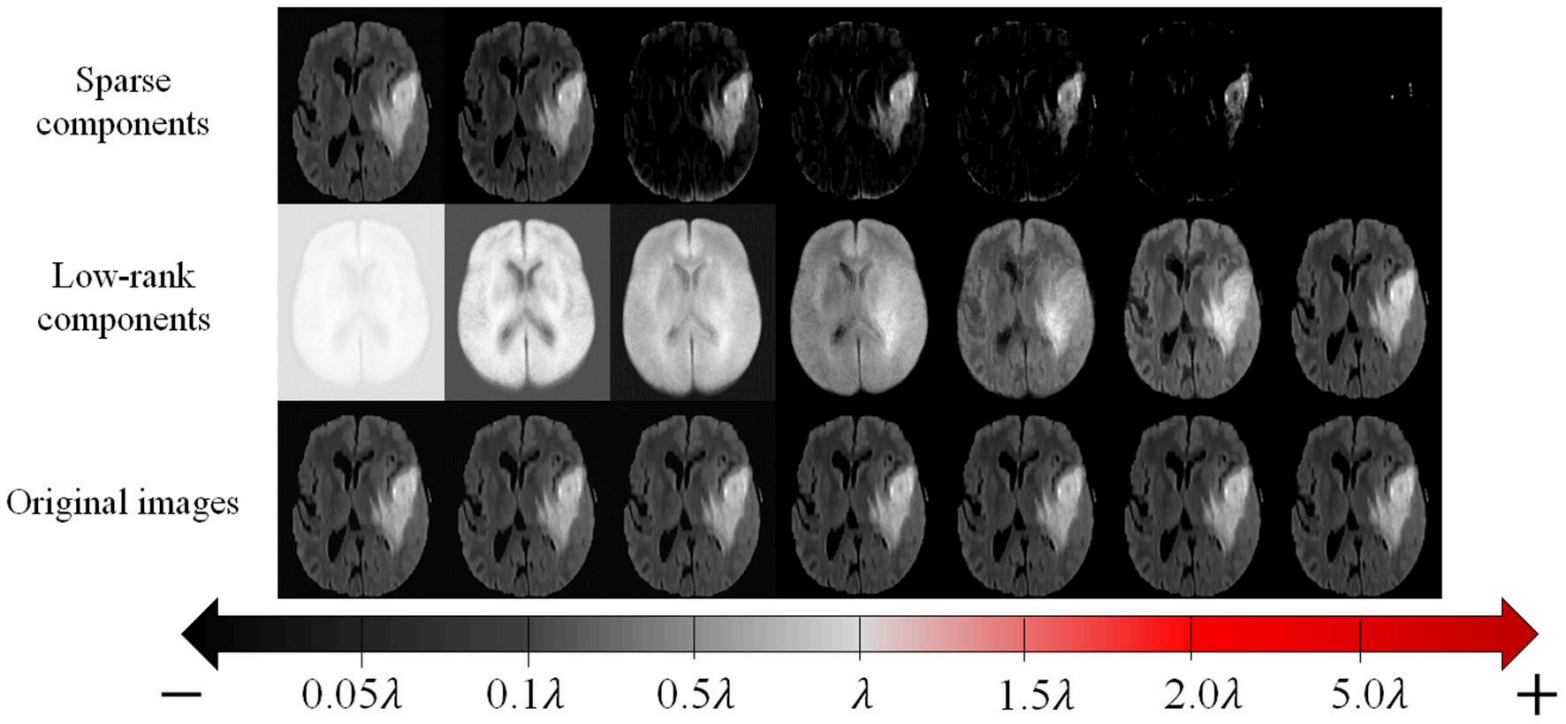
The original MR images **(bottom row)**, and corresponding recovered low-rank components **(middle row)** and sparse components **(top row)** given by conventional LSD method, with different values of regularizing parameter λ.

To realize practical and reliable recovery and segmentation of pathological MR images, the LSD remains three issues to be addressed: (1) all images shall be aligned in the same spatial domain; (2) *S* shall be structured sparse to better represent the structured sparsity of the contiguous tumor region in the whole image; (3) as illustrated in Figure 3, as the parameter λ becomes smaller, the low-rank images can recover tumor regions more reliably, but also generate more blurred appearances in originally normal regions. Therefore, regularizing parameter λ shall be different regarding to normal and tumor regions, thus to adaptively balance the low-rank and sparse components to reliably recover tumor regions meanwhile retaining normal brain regions.

### 2.2. Transformed Low-Rank and Structured Sparse Decomposition (TLS^2^D)

To tackle the issues in LSD, we propose a transformed low-rank and structured sparse decomposition. Firstly, considering the tumor region usually occupies a contiguous portion of the brain image, thus it is reasonable to model the tumor region using the structured sparsity norm. Inspired by the structured sparsity in Jia et al. (2012), we introduce a structured sparsity norm Ω(*S*) to model tumor region, and define low-rank and structured sparse decomposition (LS^2^D) as:

(2) $$\begin{array}{l} \underset{L,S}{\min}\;||L|{|}_{*}+\lambda \Omega \left(S\right)\;s.t.\;A=L+S, \end{array}$$

where

(3) $$\begin{array}{l} \Omega \left(S\right)=\overset{n}{\underset{i=1}{\sum }}\underset{g\in G}{\sum }||mat{\left(S_{i}\right)}_{g}|{|}_{\infty }. \end{array}$$

In Equation (3), $S_{i}\in {\mathbb{R}}^{m}$ is the *i*^*th*^ column in *S*; $mat\left(S_{i}\right)\in {\mathbb{R}}^{w\times h}$ is the matrix form of *S*_*i*_. We define 3 × 3 overlapping-patch groups *G* in *mat*(*S*_*i*_), and *g*∈*G* represents each 3 × 3 group. Each group overlaps 6 pixels with its neighbor group. ||·||_∞_ is the ℓ_∞_ norm (i.e., the maximum value in a group *g*). The structured sparsity norm Ω(*S*) in Equation (2) can constrain *S* to be structured distribution thus better representing tumor region.

During the decomposition, the spatial mismatch between different images may cause undesired sparse noise. To alleviate the spatial mismatch, we perform image alignment in our decomposition procedure (Zheng et al., 2017). The proposed TLS^2^D is defined as follows:

(4) $$\begin{array}{l} \underset{L,S,\tau }{\min}\;||L|{|}_{*}+\lambda \Omega \left(S\right)\;s.t.\;A◦\tau =L+S, \end{array}$$

where τ denotes a set of *n* affine transformations τ_1_, τ_2_, …, τ_*n*_ that warps *A* to align all images; $A◦\tau =\left[vec\left(A_{1}◦\tau_{1}\right),vec\left(A_{2}◦\tau_{2}\right),\ldots ,vec\left(A_{n}◦\tau_{n}\right)\right]\in {\mathbb{R}}^{m\times n}$.

The optimization of our TLS^2^D in Equation (4) is non-convex and difficult to solve directly due to the nonlinearity of the τ. To tackle this issue, we can iteratively linearize about the estimate of τ according to Boyd et al. (2011) and Wang et al. (2018). Specifically, we linearize the constraint by using the local first order Taylor approximation for each image as $A◦\left(\tau +\Delta \tau \right)\approx A◦\tau +\overset{n}{\underset{i=1}{\sum }}J_{i}\Delta \tau_{i}\epsilon_{i}\epsilon_{i}^{T}$, where $\Delta \tau =\left[\Delta \tau_{1},\Delta \tau_{2},\ldots ,\Delta \tau_{n}\right]\in {\mathbb{R}}^{p\times n}$, and each $\Delta \tau_{i}\in {\mathbb{R}}^{p}$ is defined by *p* parameters of the transformation; $J_{i}=\frac{\partial }{\partial \zeta }vec\left(A_{i}◦\zeta \right){|}_{\zeta =\tau_{i}}\in {\mathbb{R}}^{m\times p}$ is the Jacobian of the image *A*_*i*_ with respect to the transformation τ_*i*_, and {ϵ_*i*_} denotes the standard basis for ℝ^*n*^. Thus, Equation (4) can be relaxed into the following optimization:

(5) $$\begin{array}{l} \underset{L,S,\Delta \tau }{\min}\;||L|{|}_{*}+\lambda \Omega \left(S\right)\;s.t.\;A◦\tau +\overset{n}{\underset{i=1}{\sum }}J_{i}\Delta \tau_{i}\epsilon_{i}\epsilon_{i}^{T}=L+S. \end{array}$$

Then the resulting convex programming in Equation (5) can be solved by ALM method (Lin et al., 2010). We formulate the following augmented Lagrangian function:

(6) $$\begin{array}{c} L\left(L,S,\Delta \tau ,Y;\mu \right)=||L|{|}_{*}+\lambda \Omega \left(S\right)+\langle Y,h\left(L,S,\Delta \tau \right)\rangle \\ +\frac{\mu }{2}||h\left(L,S,\Delta \tau \right)|{|}_{F}^{2}, \end{array}$$

where $h\left(L,S,\Delta \tau \right)=\;A◦\tau +\overset{n}{\underset{i=1}{\sum }}J_{i}\Delta \tau_{i}\epsilon_{i}\epsilon_{i}^{T}-L-S$; *Y*∈ℝ^*m* × *n*^ is the Lagrangian multiplier and μ is a positive hyperparameter; 〈·, ·〉 denotes the matrix inner product, and ||·||_*F*_ is the Frobenius norm. The ALM algorithm then estimates both the optimal solution and the Lagrange multiplier by iteratively solving the following four subproblems:

(7) $$\begin{array}{l} \begin{array}{cc} L^{t+1} & =\arg\underset{L}{\min}L\left(L,S^{t},\Delta \tau^{t},Y^{t};\mu^{t}\right),\\ S^{t+1} & =\arg\underset{S}{\min}L\left(L^{t+1},S,\Delta \tau^{t},Y^{t};\mu^{t}\right),\\ \Delta \tau^{t+1} & =\arg\underset{\Delta \tau }{\min}L\left(L^{t+1},S^{t+1},\Delta \tau^{t},Y^{t};\mu^{t}\right),\\ Y^{t+1} & =Y^{t}+\mu^{t}h\left(L^{t+1},S^{t+1},\Delta \tau^{t+1}\right), \end{array} \end{array}$$

where superscript *t* denotes the iteration. In each iteration, the first problem in Equation (7) can be expressed as

(8) $$\begin{array}{l} L^{t+1}=\arg\underset{L}{\min}\;\left\{||L|{|}_{*}+\frac{\mu^{t}}{2}||H_{L}-L|{|}_{F}^{2}\right\}, \end{array}$$

where $H_{L}=A◦\tau +\overset{n}{\underset{i=1}{\sum }}J_{i}\Delta \tau_{i}^{t}\epsilon_{i}\epsilon_{i}^{T}-S^{t}+Y^{t}/\mu^{t}$. The problem in Equation (8) has a simple closed-form solution by soft thresholding operator (Parikh et al., 2014). Suppose the singular value decomposition of *H*_*L*_ is (*U*, Σ, *V*) = *svd*(*H*_*L*_), then $L^{t+1}=US_{\frac{1}{\mu^{t}}}\left[\Sigma \right]V^{T}$, where $S_{\frac{1}{\mu }}\left(x\right)=\left\{{\left[x-\frac{1}{\mu }\right]}_{+}-{\left[-x-\frac{1}{\mu }\right]}_{+}\right\}$ is the soft thresholding operator and [·]_+_ = *max*(·, 0).

The second problem in Equation (7) can be rewritten as

(9) $$\begin{array}{l} S^{t+1}=\arg\underset{S}{\min}\;\left\{\frac{\mu^{t}}{2}||H_{S}-S|{|}_{F}^{2}+\lambda \Omega \left(S\right)\right\}, \end{array}$$

where $H_{S}=A◦\tau +\overset{n}{\underset{i=1}{\sum }}J_{i}\Delta \tau_{i}^{t}\epsilon_{i}\epsilon_{i}^{T}-L^{t+1}+Y^{t}/\mu^{t}$. The problem in Equation (9) is the proximal operator associated with the structured sparsity norm, which can be calculated by solving a quadratic min-cost flow problem (Mairal et al., 2010).

Then given the current estimated *L*^*t*+1^ and *S*^*t*+1^, the solution of the third problem in Equation (7) can be calculated as

(10) $$\begin{array}{l} \Delta \tau^{t+1}=\overset{n}{\underset{i=1}{\sum }}J_{i}^{†}\left(L^{t+1}+S^{t+1}-A◦\tau -Y^{t}/\mu^{t}\right)\epsilon_{i}\epsilon_{i}^{T}, \end{array}$$

where $J_{i}^{†}$ denotes the Moore-Penrose pseudoinverse of *J*_*i*_. We summarize the solver for Equation (4) in Algorithm 1.

**Algorithm 1:** Transformed Low-Rank and Structured Sparse Decomposition (TLS^2^D)

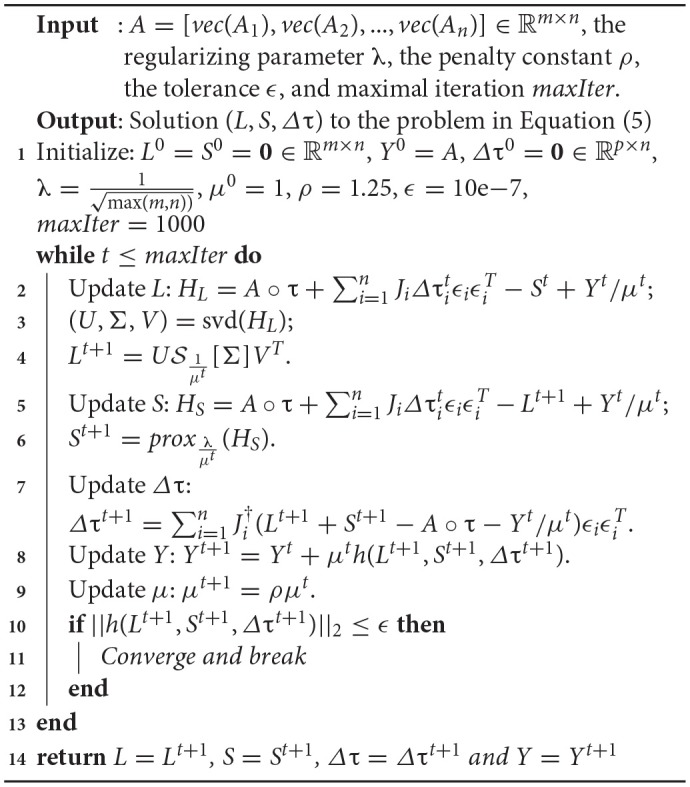

### 2.3. Recovery and Segmentation Framework

In our recovery and segmentation framework, at the first step we employ the proposed TLS^2^D to align all MR images and meanwhile obtaining low-rank and structured sparse images (see Figure 2). The low-rank images at this step blur the tumor region and yet cannot reliably recover the normal image appearances. To address this problem, we propose to leverage the obtained structured sparse component to adjust the regularizing parameter λ in Equation (4) for the adaptive sparsity constraint.

Specifically, we compute the saliency maps of the MR images using (Perazzi et al., 2012). The saliency map indicates the saliency of each pixel to catch the human attention, with value 1 denoting the highest attention and 0 denoting no attention. According to (Perazzi et al., 2012), in order to calculate the saliency of an image, we first abstract this image into perceptually homogeneous elements using (Achanta et al., 2012). We then employ a set of high-dimensional Gaussian filters (Adams et al., 2010) to calculate two contrast measures (i.e., the uniqueness and spatial distribution of elements), and use these two measures to predict the final saliency of each pixel. In pathological MR images, the most salient part shall be the tumor regions. We then obtain the tumor probability map of an image by computing the dot product between its binary structured sparse image and its corresponding saliency map, as shown in Figure 2. The tumor probability map indicates the probability of each pixel being tumor region. We denote tumor probability map $P\;=\;\left[vec\left(P_{1}\right),vec\left(P_{2}\right),\ldots ,vec\left(P_{n}\right)\right]\in {\mathbb{R}}^{m\times n}$.

Finally, we use the tumor probability map to adaptively adjust the regularizing parameter λ in Equation (4). We define the adaptive TLS^2^D to obtain the final tumor segmentation and well recovered quasi-normal images:

(11) $$\begin{array}{l} \underset{L,S,\tau }{\min}\;||L|{|}_{*}+\lambda \left(1-P\right)⊙\Omega \left(S\right)\;s.t.\;A◦\tau =L+S, \end{array}$$

where **1** ∈ ℝ^*m*×*n*^, with each element equals to 1. λ(**1** − *P*) is the adaptive regularizing matrix. ⊙ denotes dot product. In such a way, the sparse constraints for tumor and normal regions are set differently, thus our TLS^2^D can reliably recover tumor regions meanwhile retaining normal regions.

## 3. Experiments and Results

The proposed TLS^2^D method was evaluated on both synthetic and real MR brain tumor images. We also extensively compared our method with state of the art, including Robust Principal Component Analysis (RPCA) (Candès et al., 2011), Robust Alignment by Sparse and Low-rank decomposition (RASL) (Peng et al., 2012), and Spatially COnstraint LOw-Rank (SCOLOR) (Tang et al., 2018). Specifically, the RPCA method is one of the most classical and successful low-rank and sparse decomposition schemes; the RASL method considers spatial mismatch between different images and hence adds image alignment into the low-rank based decomposition procedure; the SCOLOR method imposes spatial constraint on sparse component to restrict its structured sparsity.

The metrics employed to quantitatively evaluate recovery and segmentation performance was structural similarity index (SSIM) (Wang et al., 2004) and Dice index (Chang et al., 2009), respectively. The SSIM index is the most popular metric to evaluate the similarity of two images by using structural information. The SSIM of two images *x* and *y* is:

(12) $$\begin{array}{l} SSIM\left(x,y\right)=\frac{\left(2\mu_{x}\mu_{y}+c_{1}\right)\left(2\sigma_{xy}+c_{2}\right)}{\left(\mu_{x}^{2}+\mu_{y}^{2}+c_{1}\right)\left(\sigma_{x}^{2}+\sigma_{y}^{2}+c_{2}\right)}, \end{array}$$

where μ_*x*_ and μ_*y*_ is the average of *x* and *y*; σ_*x*_ and σ_*y*_ is the variance of *x* and *y*, respectively; σ_*xy*_ is the covariance of *x* and *y*; *c*_1_ and *c*_2_ are two constants to stabilize the division. The Dice index is used for comparing the similarity of two regions, and can be calculated as:

(13) $$\begin{array}{l} Dice=\frac{2|G\cap T|}{\left|G|+|T\right|}, \end{array}$$

where *T* and *G* denotes the segmented tumor region and ground truth, respectively.

### 3.1. Validation on Synthetic MR Brain Tumor Images

**Figure 4:**
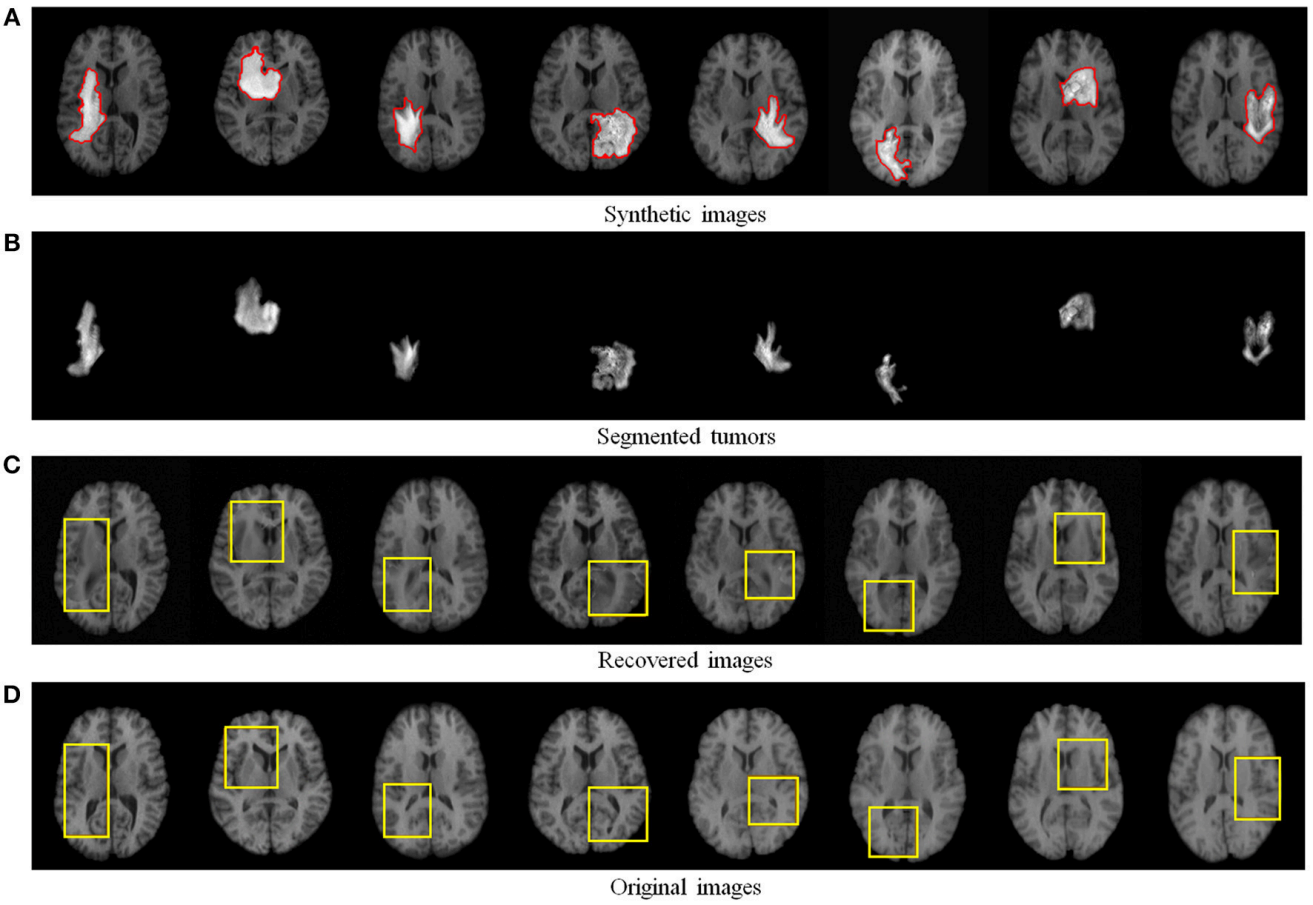
Recovery and segmentation of **(A)** synthetic MR brain tumor images: **(B)** the segmented tumors, **(C)** the recovered images with normal brain appearances, **(D)** the corresponding original MR images from LPBA40 (Shattuck et al., 2008). Yellow boxes illustrate the reliably recovered regions.

We first quantitatively evaluated the recovery performance of our method on synthetic tumor images. The synthetic MR brain tumor images are based on images from a public dataset LPBA40 (Shattuck et al., 2008). The LPBA40 dataset includes 40 T1-weighted MR normal brain images. Some example normal images from LPBA40 are shown in Figure 4D. We generated the synthetic tumor images by fusing tumor regions derived from a real MR tumor image dataset BRATS2018 (Menze et al., 2015) (see Figure 4A).

**Figure 5:**
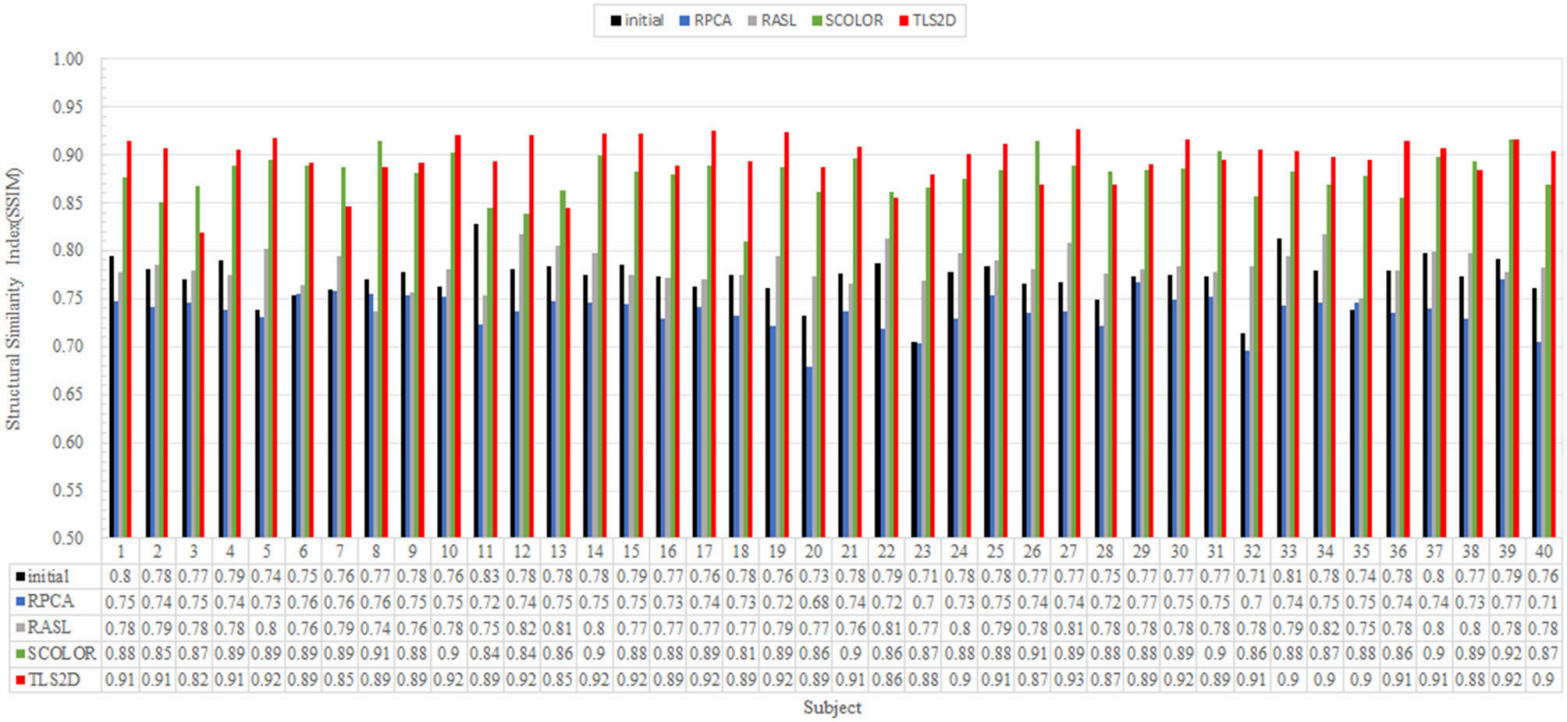
The structural similarity index (SSIM) between each of the original MR images and the corresponding recovered images by different methods. The “Initial” indicates the SSIM between the synthetic tumor images and the corresponding original images.

Figure 4 visualizes some recovery and segmentation results obtained by our method. It can be observed that our method can reliably extract the tumor regions, and recover these regions with normal brain appearances. Figure 5 further illustrates the quantitative SSIM values between the original MR images and the recovered images by different methods. Our TLS^2^D method consistently achieves the most similar image appearance to the original images from LPBA40. In addition, Table 1 lists the Dice indices of the segmented tumor regions by different methods. Our TLS^2^D achieves the best segmentation performance.

**Table 1:** Dice values of different methods on synthetic and real MR brain tumor images.

**Data type**	**RPCA**	**RASL**	**SCOLOR**	**TLS^**2**^D (Ours)**
Synthetic tumor images	0.54 ± 0.25	0.62 ± 0.26	0.70 ± 0.26	**0.80**±**0.28**
Real tumor images	0.46 ± 0.20	0.51 ± 0.20	0.63 ± 0.30	**0.75**±**0.26**

*Best results are highlighted in bold*.

### 3.2. Evaluation on Real MR Brain Tumor Images

**Figure 6:**
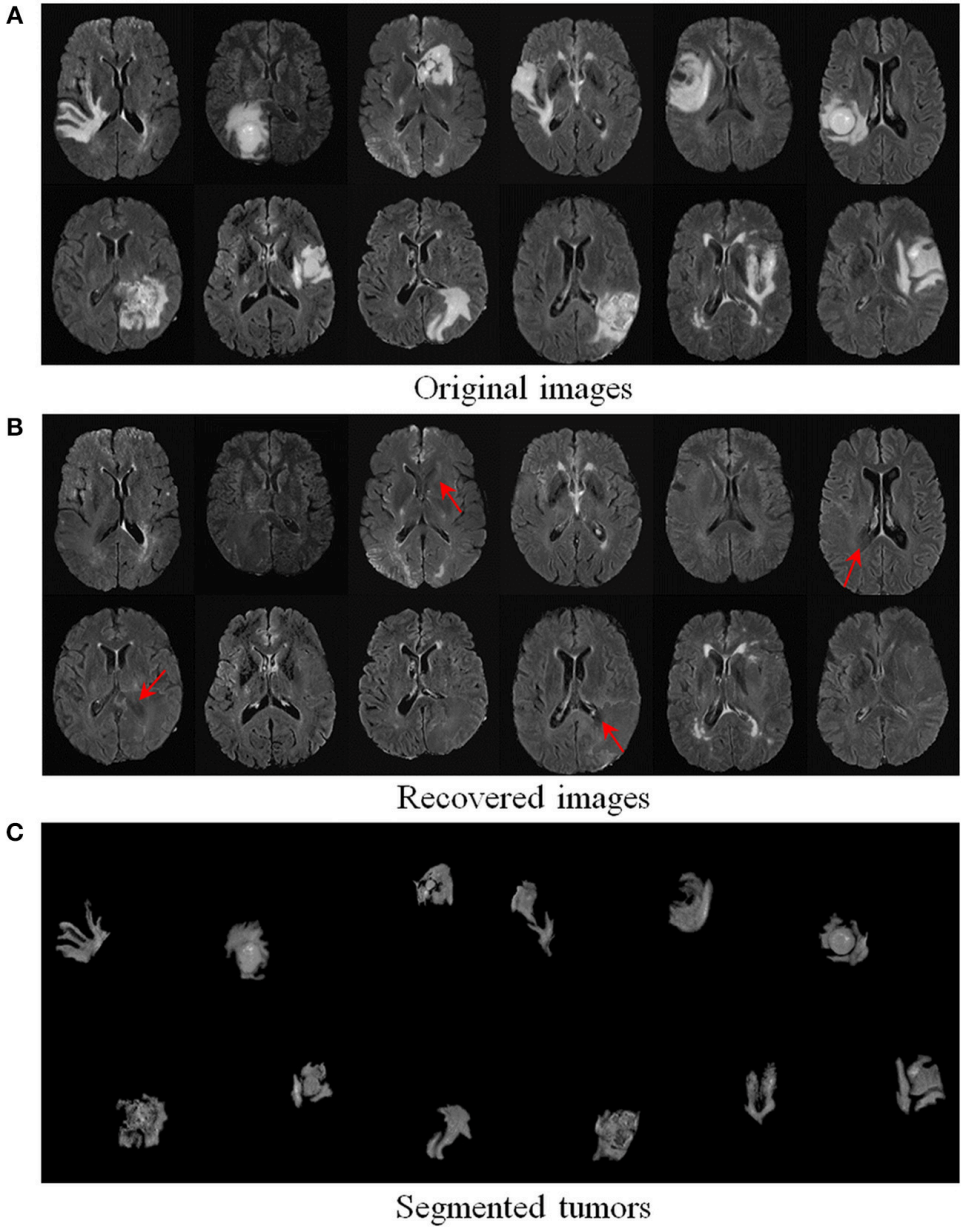
Recovery and segmentation of **(A)** real MR brain tumor images from BRATS2018 (Menze et al., 2015); **(B)** the recovered images with normal brain appearances, **(C)** the tumor segmentation results. Red arrows indicate well recovered brain structures.

We further evaluated the efficacy of our method on 124 real T2-weighted FLAIR MR brain tumor images from the dataset BRATS2018 (Menze et al., 2015). Table 1 demonstrates that our TLS^2^D method achieves the best tumor segmentation results. Figure 6 illustrates some example recovery and segmentation results obtained by our method. It can be seen from Figure 6 that our method can achieve satisfactory recovery and segmentation performance. The recovered images by our method could infer the plausible brain structures, see red arrows in Figure 6B.

### 3.3. Application to Multi-Atlas Segmentation

The recovery of pathological regions with normal brain appearances is beneficial for other image computing tasks, such as multi-atlas segmentation (MAS). The MAS attempts to register multiple normal brain atlases to a new brain image, thus to map their corresponding anatomical labels to the new brain image for the brain segmentation. Conventional MAS methods may not perform well when images are with tumor regions, because the appearance change induced by these regions cause difficulties in registering multiple atlases to the brain tumor image. We conducted multi-atlas segmentation based on the recovered images to demonstrate the benefit of our method on image recovery.

**Figure 7:**
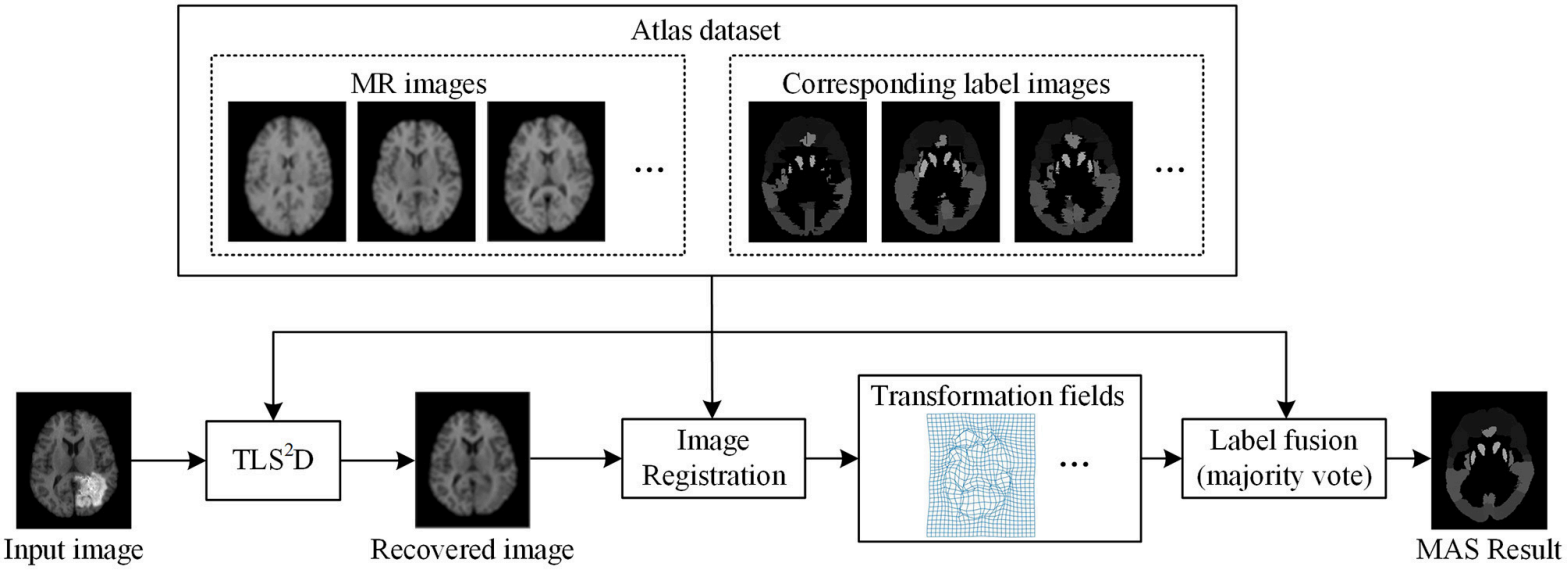
The illustration of the multi-atlas segmentation framework.

**Figure 8:**
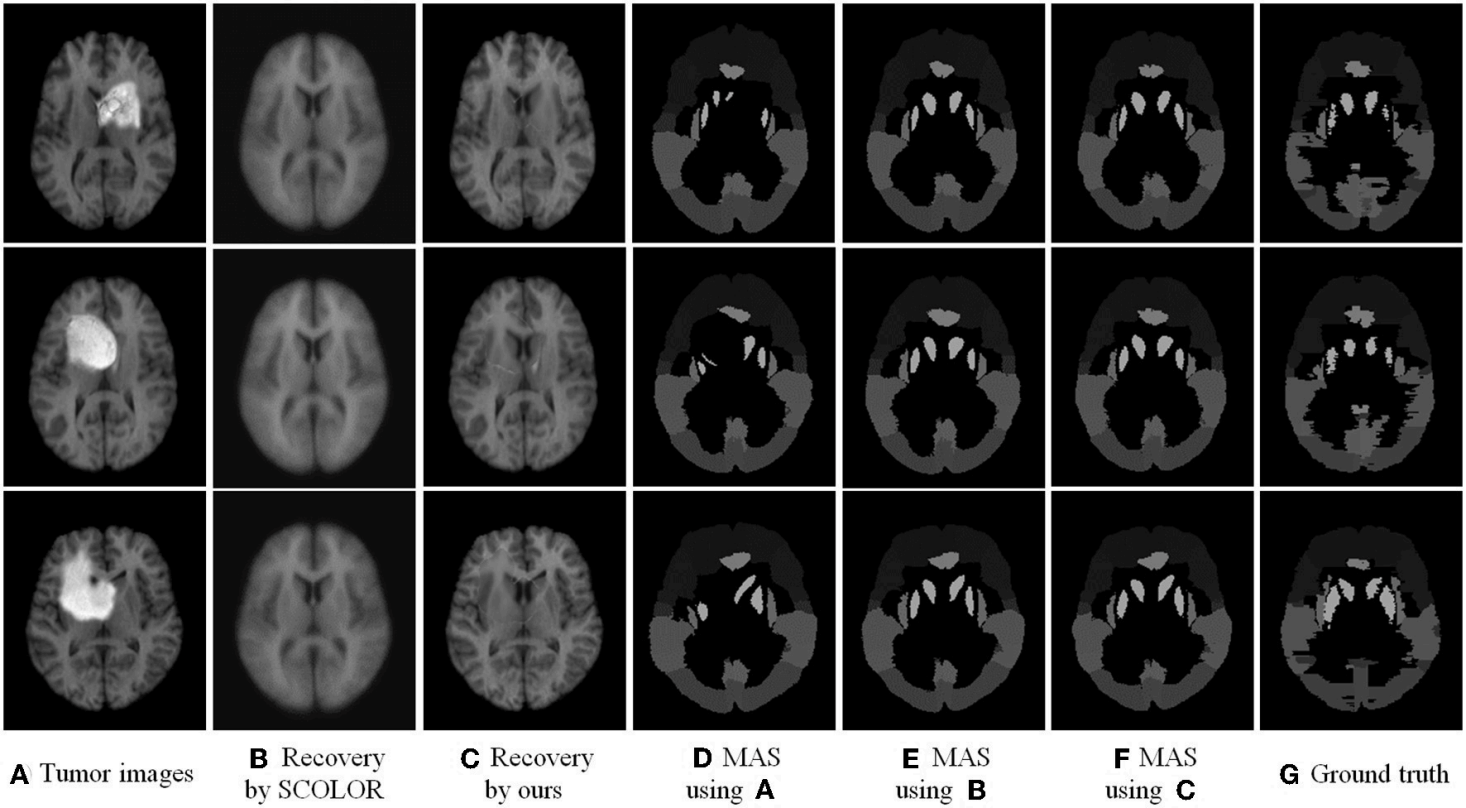
Multi-atlas segmentation results: **(A)** brain tumor images, **(B)** the recovered images by SCOLOR method, **(C)** our recovered images with quasi-normal brain appearances, **(D)** the MAS results by using original tumor images, **(E)** the MAS results by using the recovered images from SCOLOR, **(F)** the MAS results by using our recovered images, and **(G)** the segmentation ground truth.

**Figure 9:**
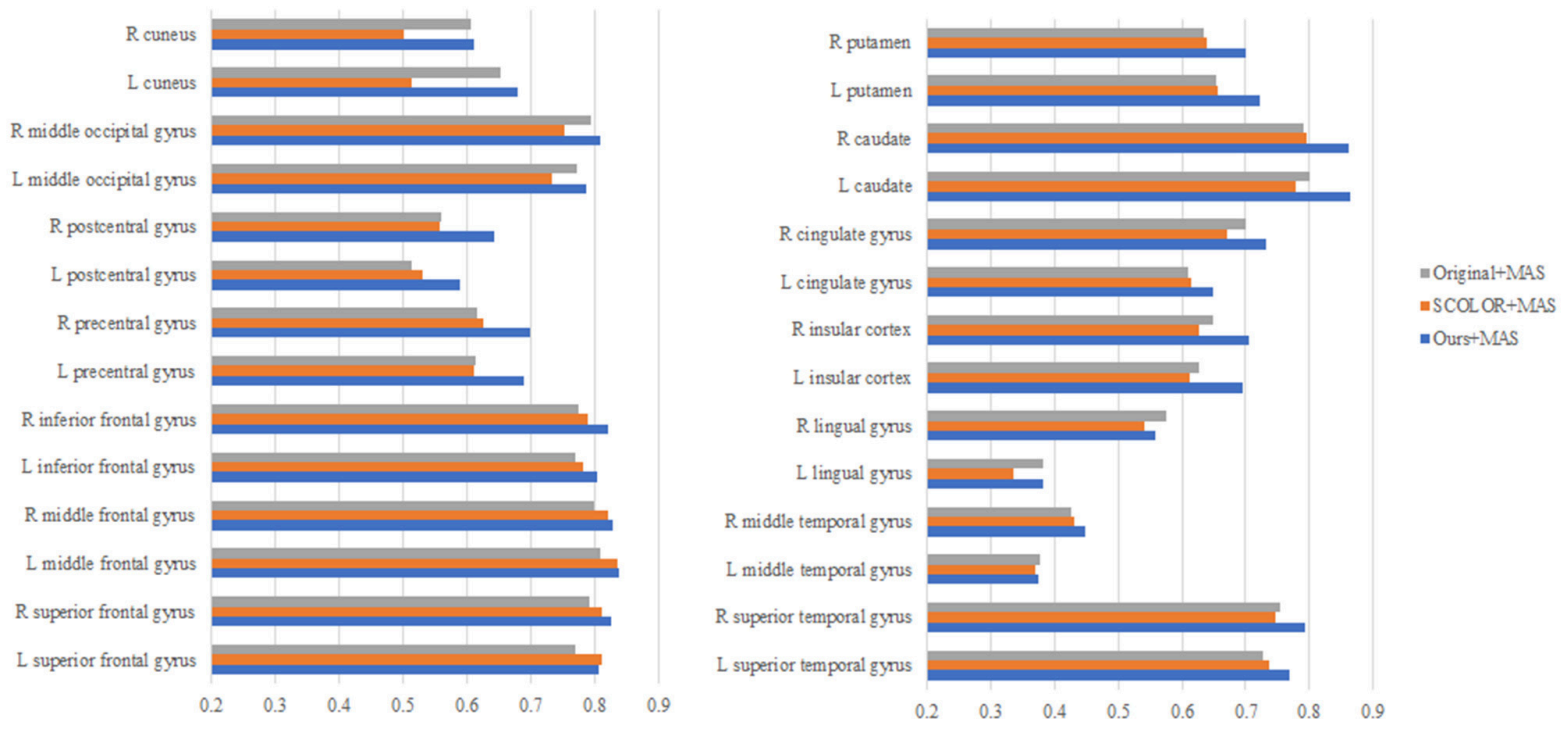
The average Dice indices of different brain regions of 40 segmented brain tumor images using MAS+original images, MAS+SCOLOR recovered images, and MAS+our recovered images, respectively.

We used 40 T1-weighted MR images and their corresponding segmentation labels from LPBA40 (Shattuck et al., 2008) to conduct MAS. For each time of MAS, we chose one image to generate synthetic tumor image, and employed the remaining 39 images as multiple atlases. As shown in Figure 7, we then used the proposed TLS^2^D method to obtain the recovered image, and utilized an intensity-based non-rigid registration method (Myronenko and Song, 2010) to map multiple atlases to the recovered image for the brain segmentation via majority vote based label fusion. Figure 8 shows some MAS results obtained by using the recovered images and original images, respectively. It can be observed that the brain segmentations using our recovered images outperform those using original tumor images, especially in the regions tumor occupied. It also can be observed from Figure 8 that compared to SCOLOR method, our method can produce much clearer recovered images. Figure 9 further illustrates the average Dice indices of different brain regions of 40 segmented brain tumor images using MAS+original images, MAS+SCOLOR recovered images and MAS+our recovered images, respectively. The MAS using our recovered images consistently achieve better Dice indices compared to the MAS using original images and recovered images from SCOLOR, which demonstrates our method is potentially useful to improve the MAS when images are with pathological regions.

## 4. Discussion and Conclusion

In this study, we have proposed a novel low-rank based method, called transformed low-rank and structured sparse decomposition (TLS^2^D), for the reliable recovery and segmentation of pathological MR brain images. By integrating the structured sparsity, image alignment, and adaptive spatial constraint into a unified matrix decomposition framework, our method is robust for extracting pathological regions, and also is reliable for recovering quasi-normal MR appearances. The recovered image is beneficial for subsequent image computing procedures, such as atlas-based segmentation. We have compared the proposed TLS^2^D method with several state-of-the-art low-rank based approaches on synthetic and real MR brain images. Regarding these compared methods, the RPCA method is a conventional low-rank and sparse decomposition method; the RASL method embeds image alignment into LSD framework; the SCOLOR method imposes spatial constraint on sparse component. Experimental results show our method consistently outperforms all compared methods, which demonstrates the contribution of the proposed transformed low-rank and structured sparse decomposition with adaptive sparse constraint on simultaneous recovery and segmentation.

Computer aided methods that can assist clinicians to analyze the MR brain tumor scans are of essential significance for improved diagnosis, treatment planning and patients' follow-up. Automated tumor segmentation is the primary research task for analyzing the pathological images, and has been extensively investigated in the literature (Gordillo et al., 2013; Menze et al., 2015; Zhou et al., 2017). However, in addition to tumor segmentation task, the presence of pathologies in MR images poses challenges in other image computing tasks, such as intensity-/feature-based image registration (Sotiras et al., 2013) and atlas-based segmentation of brain structures (Cabezas et al., 2011), due to the structure and appearance changes of pathological brain images. Thus the recovery of pathological regions with normal brain appearances is beneficial for most image computing procedures. To this end, we consider to integrate the registration, segmentation and recovery procedures into a unified decomposition framework. The proposed TLS^2^D is a generic method for analyzing the MR brain tumor scans. It is worth noting that although our method is able to provide recovered images with quasi-normal brain appearances, the recovered regions may have some artifacts, located in the region around original tumor boundary, as shown in Figure 8. This is mainly due to the distinction of sparse constraints between inner boundary (tumor region) and outer boundary (normal region). Even so, compared to the original pathological images, our recovered images are more similar to the normal brain images, thus are more convenient to be used for other image computing tasks, such as multi-atlas segmentation shown in section 3.3.

The tumor region usually occupies a contiguous portion in the MR brain image, thus the distributions of tumor pixels are not pixel-wised sparse but structurally sparse. This motivates us to model the tumor region using the structured sparsity norm. Considering that the structured sparsity norm described in Jia et al. (2012) can effectively encourage sparse component to distribute in structured patterns and also its facility to be implemented in the low-rank and sparse decomposition scheme, we employ this structured sparsity norm (Jia et al., 2012) to model tumor region in this study. Note that the structured sparsity (Jia et al., 2012) could be replaced by sparsity in a different basis (e.g., a wavelet basis), but such sparsity needs to take into account the spatial connection of the sparse pixels.

The tumor segmentation performance of our method still could be improved, especially compared with the state-of-the-art deep learning based segmentation models (Pereira et al., 2016; Havaei et al., 2017). However, these deep learning based methods typically require enormous amount of high-quality labeled images to train a model for medical image segmentation. Although some recent approaches (Mlynarski et al., 2018; Shah et al., 2018) proposed a mixed-supervision scheme, which employed a minority of images with high-quality per-pixel labels and a majority of images with coarse-level annotations (bounding boxes, landmarks or image-level annotations) to train the deep neural networks; preparing annotations such as bounding boxes and landmarks is still laborious. Compared with deep learning based methods, our advantage is that the proposed TLS^2^D does not require labeled images to train a segmentation model; it extracts tumor regions by analyzing normal MR image appearances from unlabeled population data. What's more, the segmentation results of our method can alleviate the image labeling procedure by the clinicians. Our segmentation results could further be used as label information for the semi-supervised training of deep learning based segmentation models (Papandreou et al., 2015; Bai et al., 2017).

## Author Contributions

CL, YW, TW, and DN: response for study design; CL: implemented the research and conducted the experiments; CL and YW: conceived the experiments, analyzed the results, and wrote the main manuscript text and prepared the figures. All authors reviewed the manuscript.

### Conflict of Interest Statement

The authors declare that the research was conducted in the absence of any commercial or financial relationships that could be construed as a potential conflict of interest.
